# Investigation of the Use of Circulating Long Non-coding RNA HOXA Transcript at the Distal Tip (LncRNA HOTTIP) as a Biomarker in Breast Cancer

**DOI:** 10.7759/cureus.50019

**Published:** 2023-12-06

**Authors:** Ioannis I Psathas, Konstantinos Birbas, Gerasimos Bonatsos, Romanos Trantas, Louisa G Mahaira, Ioannis Kaklamanos

**Affiliations:** 1 Surgical Oncology, General Oncological Hospital of Kifissia "Agioi Anargyroi", Athens, GRC; 2 Surgery, General Oncological Hospital of Kifissia "Agioi Anargiri" / National and Kapodistrian University of Athens, Athens, GRC; 3 Nursing School, National and Kapodistrian University of Athens, Athens, GRC; 4 Genetics, “Saint Savvas” General Anti-Cancer and Oncological Hospital of Athens, Athens, GRC; 5 Surgical Oncology, National and Kapodistrian University of Athens, Athens, GRC

**Keywords:** prognostic markers, molecular biomarker, breast cancer research, breast cancer biology, lncrna

## Abstract

The critical need for new diagnostic and prognostic methods is highlighted by the fact that breast cancer continues to be the top cause of cancer-related deaths globally. Due to the dysregulation of long non-coding RNAs (lncRNAs) in numerous malignancies, they have become potential biomarkers in cancer. Recent research has focused on the lncRNA HOTTIP (HOXA transcript at the distal tip), which has a function in breast cancer metastasis and carcinogenesis. Until recently, HOTTIP had only been measured in cancer tissues and specimens. The aim of this study is to assess the amounts of the lncRNA HOTTIP in the blood serum of 46 breast cancer patients using real-time PCR analysis and identify the relationships between HOTTIP expression and several known clinical and pathological factors, including tumor grade, stage, lymph node involvement, hormone receptor status, and cell proliferation. The results of the study confirmed a positive relation of HOTTIP expression and breast cancer aggressiveness and metastatic behavior. The analysis results showed elevated HOTTIP values in stage III and T3/T4 tumors with multifocal characteristics and in lymph node involvement. Our findings raise the possibility of HOTTIP serving as a future prognostic biomarker for breast cancer patients.

## Introduction

Being the most prevalent cancer in women globally, breast cancer poses a serious threat to global health [1,2]. For a successful course of therapy and better patient outcomes, early identification of breast cancer is essential [3]. Despite improvements in screening and diagnostic methods for early breast cancer detection, more accurate and trustworthy biomarkers are still required for the diagnosis, prognosis, and follow-up of this illness [4,5,6,7].

Non-coding RNAs (ncRNAs) have attracted a great deal of interest in recent years because of their function in cancer biology [8,9,10,11,12]. In several malignancies, including breast cancer, long non-coding RNAs (lncRNAs), a subclass of ncRNAs, have become potential biomarkers and therapeutic targets [13,14,15]. HOTTIP (HOXA transcript at the distal tip), one of the many lncRNAs investigated, has demonstrated potential as a possible biomarker for breast cancer [14,16,17].

The HOXA locus is the source of the well-studied lncRNA HOTTIP, which has been linked to several cellular functions, such as cell division, migration, and proliferation [15,18,19]. In addition, a growing body of research indicates that dysregulated HOTTIP expression is linked to the onset and progression of disease, particularly breast cancer [18]. This makes HOTTIP an attractive molecule to research for its possible application in the detection and treatment of breast cancer [20].

The purpose of this study is to conduct a thorough examination of the usage of circulating HOTTIP as a breast cancer biomarker. We will talk about the dysregulation of HOTTIP in breast cancer, the current knowledge of its molecular roles, and its potential as a non-invasive diagnostic and prognostic tool. We will also go through the difficulties and possibilities of using circulating lncRNAs as biomarkers in clinical practice, including concerns like sensitivity, specificity, and standardization of detection techniques [21].

Through this research, we want to add to the expanding concept of use of ncRNAs in breast cancer and maybe progress the creation of new and improved methods for the early identification and treatment of this common and deadly condition [5,22,23].

## Materials and methods

Blood samples from 46 patients with breast cancer were collected, one day prior to surgery, at Kifissia General Oncological Hospital “Agioi Anargiroi” in Kifissia, Greece, between June 2017 and July 2018. Serum was isolated following centrifugation of the samples and was stored at −80 °C until further analyzed. Peripheral blood samples were also obtained from healthy volunteers. Aliquots of sera from these individuals were kept frozen and used as the control group. The diagnosis of breast cancer in the first group had been established through fine needle aspiration (FNA) or core biopsy.

Total RNA was extracted from the serum using the Qiagen miRNeasy Serum/Plasma kit (Qiagen, Hilden, Germany) according to the manufacturer’s instructions. cDNA was generated with the SuperScript™ First-Strand Synthesis System for RT-PCR (Thermo Fisher Scientific, USA) and used as template for real-time PCR. Real-time PCR analysis was performed using Invitrogen™ Platinum™ Quantitative PCR SuperMix-UDG w/ROX and TaqMan probe chemistry, on an ABI 7500 PCR machine (Applied Biosystems™, USA). For the detection of lncRNA HOTTIP, we used commercially available, predesigned primers - TaqMan probe set (Hs00955374_s1, Thermo Fisher Scientific). The levels of glyceraldehyde-3-phosphate dehydrogenase (GAPDH) were estimated in the same samples using the respective predesigned primers - TaqMan probe set (Hs03929097_g1, Thermo Fisher Scientific) - and served as a housekeeping gene. The delta-delta Ct (DDCt) method was used for data analysis and HOTTIP serum-level estimation (Table 1). After the surgical procedure (mastectomy, lumpectomy, sentinel lymph node dissection (SLND), and lymphadenectomy), the specimens were sent for pathological examination. Immunohistochemistry procedure was performed in all 46 samples following the streptavidin/biotin technique. In all specimens, the estrogen receptor (ER), progesterone receptor (PR), human epidermal growth factor receptor 2 (HER2), Ki67, histological type, multifocality, degree of differentiation, staging, and axillary lymph nodes were examined. For the ER and PR measurements, the 6F11 antibody in the 1/50 title was used. Results of the HER-2 immunochemical reaction were evaluated with HercepTest (0, 1+, 2+, 3+). For the Ki-67 marker, the MIB-1 labelling index was used in the 1/50 title. The TNM system (American Joint Committee on Cancer (AJCC)) was used for the evaluation of the tumor size and number of positive axillary lymph nodes.

**Table 1:** HOTTIP values (arbitrary units) In science and technology, an arbitrary unit or procedure defined unit is a relative unit of measurement that shows the ratio of amount of substance or other quantities to a predetermined reference measurement. HOTTIP: HOXA transcript at the distal tip, GAPDH: glyceraldehyde-3-phosphate dehydrogenase (used as an endogenous control for quantitative RT-PCR analysis because its expression is consistent at different time points and various experimental manipulations), CT: threshold cycle for each sample for each gene expression measured, ΔCT: CT HOTTIP - CT GAPDH, ΔΔCT: ΔCT sample - ΔCT pool, 2^(-ΔΔCT): standard strategy for qPCR data analysis based on the PCR efficiency

Samples	HOTTIP	GAPDH	ΔCT	ΔΔCT	2^(-ΔΔCT)
1	39.99	31.58	8.41	3.72	0.0758872
2	35.46	33.7	1.76	-2.93	7.621104
3	35.52	32.33	3.19	-1.5	2.8284271
4	36.01	3.2	4.01	-0.68	1.6021398
5	33.88	31.71	2.17	-2.52	5.735821
6	39.99	31.98	8.01	3.32	0.1001337
7	38.46	33.3	5.16	0.47	0.7219646
8	38.33	33.91	4.42	-0.27	1.2058078
9	37.28	32.71	4.57	-0.12	1.0867349
10	38.52	32.69	5.83	1.14	0.4537596
11	39.01	32.21	6.8	2.11	0.231647
12	37.88	33.71	4.17	-0.52	1.4339552
13	33.2	31	2.2	-2.49	5.6177795
14	35.62	32.62	3	-1.69	3.226567
15	36.93	33.7	3.23	-1.46	2.7510836
16	35.3	31.6	3.7	-0.99	1.986185
17	38.75	32.69	6.06	1.37	0.3868912
18	36.86	32.3	4.56	-0.13	1.0942937
19	34.88	31.49	3.39	-1.3	2.4622888
20	33.72	30.56	3.16	-1.53	2.8878584
21	38.34	31.38	6.96	2.27	0.2073299
22	38.94	32.47	6.47	1.78	0.2911834
23	34.36	33.41	0.95	-3.74	13.361407
24	39.31	31.9	7.41	2.72	0.1517744
25	37	32.35	4.65	-0.04	1.0281138
26	38.38	32,43	5.95	1.26	0.417544
27	39.56	32.16	7.4	2.71	0.15283
28	34.3	31.6	2.7	-1.99	3.97237
29	34.28	31.66	2.62	-2.07	4.1988667
30	38.75	32.69	6.06	1.37	0.3868912
31	38.28	32.53	5.75	1.06	0.4796321
32	36.05	31.59	4.46	-0.23	1.1728349
33	37.8	33.99	3.81	-0.88	1.8403753
34	35.17	31.16	4.01	-0.68	1.6021398
35	33.71	30.09	3.62	-1.07	2.0994334
36	39.74	31.11	8.63	3.94	0.0651541
37	38.58	32.56	6.02	1.33	0.3977682
38	37.05	32.48	4.57	-0.12	1.0867349
39	38.74	32.5	6.24	1.55	0.3415101
40	37.96	33.62	4.34	-0.35	1.2745606
41	34.4	30.79	3.61	-1.08	2.1140361
42	37.76	32.22	5.54	0.85	0.5547847
43	33.74	30.17	3.57	-1.12	2.1734697
44	38.65	32.39	6.26	1.57	0.3368084
45	38.18	32.53	5.65	0.96	0.5140569
46	39.26	32.66	6.6	1.91	0.2660925

## Results

The mean value, standard deviation, median, minimum value, and maximum value were used to present the quantitative variables. Absolute and relative frequencies were used to present the categorical variables. The Kolmogorov-Smirnov test was used to test for the normal distribution of quantitative variables.

Because of the limited variability in several categories, variables could not be applied to the associations: type of multifocality, c-erbB-2 or HER-2, and preoperative chemotherapy.

The Mann-Whitney test was used to investigate the existence of a relationship between a non-normally distributed quantitative variable and a dichotomous variable. To investigate the existence of a relationship between a quantitative variable that does not follow a normal distribution and a categorical variable, the Kruskal-Wallis test was used. Spearman's correlation coefficient was used to investigate the existence of a relationship between two quantitative variables that do not follow a normal distribution.

The two-sided level of statistical significance was set at 0.05. Data analysis was performed with IBM SPSS Statistics for Windows, version 21 (released 2012; IBM Corp., Armonk, New York, United States). The study population included 46 women with breast cancer and a mean age of 63.8 years (standard deviation = 13.3). The median age was 61 years, while the minimum and maximum ages were 37 and 91 years, respectively.

Descriptive results for the patients' clinical characteristics related to categorical variables are presented in Table 2.

**Table 2:** Descriptive results for the clinical characteristics of the patients related to categorical variables. *Staging with the TNM system does not include samples related to a part of a tumor or lymph nodes from a biopsy (core biopsy), Paget's disease, and additional tumor resection and when preoperative chemotherapy (neoadjuvant) has preceded. All patients were M0 stage. DCIS: ductal carcinoma in situ; LCIS: lobular carcinoma in situ; c-erbB-2/HER2: human epidermal growth factor receptor; T: tumor size; N: lymph nodes

Characteristics	Ν	%
Histological type		
Invasive ductal	13	28.3
Invasive ductal and DCIS	15	32.6
Invasive lobular and LCIS	6	13.0
Paget's disease of the breast nipple	1	2.2
Ductal carcinoma in situ - DCIS	2	4.3
Non-specific type - NST	9	19.6
Total	46	100
Multifocality of the tumor		
No	22	47.8
Yes	24	52.2
Type of multifocality		
Lobular carcinoma in situ - LCIS	5	20.8
Comedo type	4	16.7
Ductal carcinoma in situ - DCIS	15	62.5
Total	24	100
Degree of differentiation / histological grade		
Ι	8	17.5
ΙΙ	26	56.4
ΙΙΙ	12	26.1
Total	46	100
c-erbB-2 / HER-2		
Positive	1	2.2
Negative	45	97.8
Total	46	100
Staging *		
Τ1Ν0	12	33.3
Τ1Ν1	5	13.9
Τ2Ν0	5	13.9
Τ2Ν1	5	13.9
Τ2Ν2	1	2.8
Τ2Ν3	2	5.6
Τ3Ν1	2	5.6
Τ4Ν0	1	2.8
Τ4Ν1	1	2.8
Τ4Ν2	1	2.8
Total	35*	100
Sentinel lymph node removal - SLND		
No	23	50
Yes	23	50
Total	46	100
Sentinel positive lymph node	6/23	26.1%
Axillary lymph node disection		
No	30	65.2
Yes	16	34.8
Total	46	100
Positive axillary lymph nodes	9/16	56.2%
Overall positive lymph nodes	15/39	38.5%
Neoajvuvant chemotherapy		
No	43	93.5
Yes	3	6.5

The descriptive results for the patients' tumor biomarkers related to quantitative variables are presented in Table 3.

**Table 3:** Descriptive results for the clinical characteristics of patients related to quantitative variables. ER: estrogen receptors; PR: progesterone receptors; Ki67: cellular proliferation index

Characteristics	Average	Standard deviation	Median	Minimum value	Maximum value
Maximum diameter of the tumor (cm)	2.39	1.42	2	0,50	7
ER (%)	61.25	37.61	80	0	100
PR (%)	35.41	36.74	12.5	0	100
Ki67 (%)	19.18	17.85	12	2	95
HOTTIP	2,0567	2,6520	1,1893	0,0651	13,3614

The relationships between HOTTIP and the tumor characteristics of the patients regarding quality variables are presented in Table 4, and no statistically significant relationships were found. However, the following differences are noted: the median HOTTIP value was higher in patients with tumor multifocality. The median HOTTIP value was higher in patients with stage III cancer. The median HOTTIP value was higher in patients with T3/T4 size cancer. The median HOTTIP value was higher in patients who did not have a sentinel lymph node removed. The median HOTTIP value was higher in patients who underwent lymph node dissection.

**Table 4:** Relationships between HOTTIP and clinical characteristics of patients related to qualitative variables. * Patients that SLN dissection was performed irrelevant of the pathology result. ** Both after the positive frozen section biopsy and primarily axillary lymph node clearance. α: Mann-Whitney test, β:  Kruskal-Wallis test

Characteristics	Median HOTTIP	Interquartile range	P-value
Multifocality of the tumor			0.82^α^
No	1.1463	2.1881	
Yes	2.3281	2.4323	
Tumor grade			0.24^β^
Ι	1.2745	1.9067	
ΙΙ	1.0867	2.1589	
ΙΙΙ	2.0798	3.0182	
Tumor size			0.62^β^
Τ1	1.0867	2.5413	
Τ2	1.1173	4.9157	
Τ3/Τ4	1.8037	2.4906	
Sentinel lymph node*			0.36^α^
No	1.3543	2.3016	
Yes	0.7914	1.9737	
Axillary lymph node removal**			0.07^α^
No	1.0867	1.8083	
Yes	2.1734	2.7744	

The relationships between HOTTIP and the clinical characteristics of the patients regarding quantitative variables are presented in Table 5, and no statistically significant relationships were found. However, the following correlations are noted: increase in the progesterone receptor rate was related to the HOTTIP decrease. An increase in the lymph node positivity rate was associated with an increase in the HOTTIP.

**Table 5:** Relationships between the HOTTIP and clinical characteristics of patients related to quantitative variables. HOTTIP: HOXA transcript at the distal tip, ER: estrogen receptor, PR: progesterone receptor

Characteristics	Spearman correlation coefficient	p-value
Maximum diameter of the tumor	0.02	0.88
ER (%)	-0.08	0.61
PR (%)	-0.13	0.40
Ki67 (%)	0.07	0.67
Sentinel lymph node positivity rate (%)	-0.10	0.74
Lymph node positivity rate (%)	0.13	0.65

## Discussion

The results of this study provide important light on the association between HOTTIP expression and clinical and biological characteristics in breast cancer patients. Despite the lack of statistical significance, the data showed a number of interesting patterns that may offer insights on HOTTIP's possible contribution to the development of breast cancer. The purpose of this discussion is to situate these findings within the body of knowledge and examine how they can affect future studies and therapeutic use.

On chromosome 7q36.1, the lncRNA HOTTIP is known to control gene expression by epigenetic alteration. It is a crucial regulator of the HOXA gene cluster, which is essential for both healthy development and the development of cancer. Breast cancer is one among the cancers that have been linked to HOTTIP dysregulation [16,24].

The diagnostic utility of circulating HOTTIP levels in breast cancer has been examined in a few studies [25]. According to Wang et al. (2020), the blood levels of breast cancer patients had noticeably higher HOTTIP levels than that of healthy controls [26]. These results imply that HOTTIP may be a non-invasive biomarker for the early diagnosis of breast cancer HOTTIP as a prognostic biomarker [25].

In addition to its potential as a diagnostic biomarker, HOTTIP has also been investigated as a breast cancer prognostic biomarker [13]. According to a research, higher expression levels of HOTTIP have been linked to more aggressive tumor characteristics, such as greater tumor sizes, lymph node metastases, and advanced stage. Furthermore, lower overall survival and disease-free survival rates in breast cancer patients have been often associated to a higher HOTTIP expression [16,27].

The fundamental processes through which HOTTIP promotes the development of breast cancer are currently being studied. It is thought that HOTTIP works by regulating chromatin remodeling, which has an impact on the expression of genes involved in invasion, metastasis, and proliferation. To promote oncogenic processes, it may potentially interact with other ncRNAs and transcription factors [16,20,28, 29].

A possible link between HOTTIP expression and tumor aggressiveness has been suggested by the higher median HOTTIP values seen in patients with multifocality and advanced stage III cancer. Previous research has linked HOTTIP to the development and spread of tumors in a number of malignancies, including breast cancer. Although the association in this study did not achieve statistical significance, the pattern is consistent with other studies, highlighting the need for more research using bigger sample sizes to confirm these initial findings.

Elevated HOTTIP levels have been seen in patients with bigger tumors (T3/T4) and in those who have not had sentinel lymph nodes removed because of axillary nodes presence, suggesting a potential association between HOTTIP expression and tumor growth and lymphatic dissemination. This is consistent with studies that claim that HOTTIP is involved in the growth and invasion of cancer cells. These connections are consistent with the body of data, even if they were not statistically significant in our analysis, highlighting the need for substantial research to establish definitive links.

It is noteworthy to see the negative relationship between HOTTIP expression and the hormone receptor (PR) and lymph node positivity rates. A rise in PR-positive and lymph node involvement was correlated with a decline in HOTTIP levels. However, further research is necessary to fully understand the intricate interaction between HOTTIP and hormone receptors. The varied character of breast cancer may be reflected in variation in HOTTIP expression patterns, highlighting the need of taking molecular subtypes into account in future research.

According to current literature, the high expression of HOTTIP in tumor cells is associated with poor prognosis and promotes the stemness of breast cancer stem cells [12]. Although the exact mechanisms remain unclear, HOTTIP is involved in cancer stem cells modulation by sponging miR-148a-3p. As far as the investigation of circulating HOTTIP in serum of breast cancer patients is concerned, no updated literature is found since Abdelaleem et al. (2021)'s research [26]. 

Future research projects are crucial because of the limitations of our study, particularly the small sample size. The robustness and generalizability of the findings can be improved by extending the research cohort to include a more varied patient group, including multiple molecular subtypes of breast cancer. Furthermore, HOTTIP expression patterns along the illness continuum must be captured in long-term research. These studies may provide important information on the temporal changes in HOTTIP expression, which may be related to disease development, response to therapy, and patient survival.

Our findings raise the possibility of HOTTIP serving as a biomarker for aggressive breast cancer characteristics from a clinical standpoint. By incorporating HOTTIP evaluation into standard diagnostic methods, physicians may be able to better define risk stratification and identify patients who are at higher risk of illness recurrence or metastasis. Furthermore, it is crucial to comprehend the mechanisms through which HOTTIP affects breast cancer biology. In the age of individualized cancer treatment, targeted medicines intended to alter HOTTIP expression or obstruct its downstream signaling pathways may be a fresh idea.

Our findings have a wide range of possible applications in the field of translational research. Finding the precise molecular processes through which HOTTIP influences tumor behavior can open the door to the creation of targeted medicines that are specifically suited to each patient's HOTTIP expression patterns. Intricate networks that can be used for therapeutic treatments may also be revealed by examining the interactions between HOTTIP and other molecular actors in the tumor microenvironment.

## Conclusions

Our research illuminates the complex interactions between HOTTIP expression and clinicopathological traits in breast cancer. Although statistical significance could not be established, the recurring patterns in multifocality, disease stage, tumor size, hormone receptor status, and lymph node involvement point to a positive interaction between HOTTIP and the aggressiveness of breast cancer. Our work offers fascinating glimmers into the link between HOTTIP and breast cancer features, acting as a steppingstone in that direction. The recent findings lay a basis, but they also highlight the necessity for extensive, diverse research initiatives. As we continue to delve into the intricate molecular workings of breast cancer this may highlight the future use of HOTTIP.
